# Accidental Consumption of Aspartame in Phenylketonuria: Patient Experiences

**DOI:** 10.3390/nu13020707

**Published:** 2021-02-23

**Authors:** Ella Newbould, Alex Pinto, Sharon Evans, Suzanne Ford, Mike O’Driscoll, Catherine Ashmore, Anne Daly, Anita MacDonald

**Affiliations:** 1 Faculty of Health, Education & Life Sciences, Birmingham City University: City South Campus, Westbourne Road, Edgbaston, Birmingham B15 3TN, UK; ella.newbould@mail.bcu.ac.uk; 2 Birmingham Women’s and Children’s NHS Foundation Trust, Steelhouse Lane, Birmingham B4 6NH, UK; alex.pinto@nhs.net (A.P.); sharon.morris6@nhs.net (S.E.); catherine.ashmore@nhs.net (C.A.); a.daly3@nhs.net (A.D.); 3 National Society for Phenylketonuria, Purley CR8 9DD, UK; suzanne.ford@nspku.org; 4 School of Health and Education, Middlesex University, Room WG41A (Williams Building), The Burroughs Hendon, London NW4 4BT, UK; m.odriscoll@mdx.ac.uk

**Keywords:** phenylketonuria, phenylalanine, aspartame, sugar tax

## Abstract

Aspartame is a phenylalanine containing sweetener, added to foods and drinks, which is avoided in phenylketonuria (PKU). However, the amount of phenylalanine provided by aspartame is unidentifiable from food and drinks labels. We performed a cross-sectional online survey aiming to examine the accidental aspartame consumption in PKU. 206 questionnaires (58% female) were completed. 55% of respondents (*n* = 114) were adults with PKU or their parent/carers and 45% (*n* = 92) were parents/carers of children with PKU. 74% (*n* = 152/206) had consumed food/drinks containing aspartame. Repeated accidental aspartame consumption was common and more frequent in children (*p* < 0.0001). The aspartame containing food/drinks accidentally consumed were fizzy drinks (68%, *n* = 103/152), fruit squash (40%, *n* = 61/152), chewing gum (30%, *n* = 46/152), flavoured water (25%, *n* = 38/152), ready to drink fruit squash cartons (23%, *n* = 35/152) and sports drinks (21%, *n* = 32/152). The main reasons described for accidental consumption, were manufacturers’ changing recipes (81%, *n* = 123/152), inability to check the ingredients in pubs/restaurants/vending machines (59%, *n* = 89/152) or forgetting to check the label (32%, *n* = 49/152). 23% (*n*= 48/206) had been prescribed medicines containing aspartame and 75% (*n* = 36/48) said that medicines were not checked by medics when prescribed. 85% (*n* = 164/192) considered the sugar tax made accidental aspartame consumption more likely. Some of the difficulties for patients were aspartame identification in drinks consumed in restaurants, pubs, vending machines (77%, *n* = 158/206); similarities in appearance of aspartame and non-aspartame products (62%, *n* = 127/206); time consuming shopping/checking labels (56%, *n* = 115/206); and unclear labelling (55%, *n* = 114/206). These issues caused anxiety for the person with PKU (52%, *n* = 106/206), anxiety for parent/caregivers (46%, *n* = 95/206), guilt for parent/carers (42%, *n* = 87/206) and social isolation (42%, *n* = 87/206). It is important to understand the impact of aspartame and legislation such as the sugar tax on people with PKU. Policy makers and industry should ensure that the quality of life of people with rare conditions such as PKU is not compromised through their action.

## 1. Introduction

Aspartame, a non-nutritive sweetener, is one of the most widely used artificial sweeteners and accounts for 62% of the artificial sweetener market [1]. It is a synthetic dipeptide known as N-L-alpha-aspartyl-L-phenylalanine methyl ester (C_14_H_18_N_2_O_5_) and was accidentally discovered in 1965 [2,3]. Aspartame is completely hydrolysed to phenylalanine (50%), aspartic acid (40%) and methanol (10%) in the intestinal lumen and is rapidly metabolised by esterases and peptidases [4,5]. It is around 200 times sweeter than sucrose and it is estimated that it is added to >6000 foods and drinks [6,7]. Aspartame is approved in more than 90 countries and its safety has been evaluated by the Joint FAO/WHO Expert Committee on Food Additives (JECFA), as well as by numerous national food safety authorities, including the US Food and Drug Administration (FDA) and the European Food Safety Authority (EFSA) [8,9,10]. Aspartame can be safely consumed by healthy individuals, but it has long been recognised as a hazard to individuals with phenylketonuria (PKU) and therefore, it should be avoided [11]. The amount of phenylalanine in aspartame containing foods and drinks is not declared on ingredient labels and its impact on metabolic control in patients with PKU is not well established [12,13,14].

PKU, an autosomal recessive inherited condition, is caused by mutations in the gene encoding phenylalanine hydroxylase. It is estimated to affect 0.45 million individuals worldwide, with a global prevalence of 1:23,930 live births [15]. A rigorous lifelong low-phenylalanine diet is the principal treatment option. It requires the avoidance of high protein foods such as meat, fish, eggs, lentils, nuts, soya, bread, pasta and cheese. Daily dietary phenylalanine intake is calculated, measured and continually controlled according to individual tolerance. Eighty per cent of patients tolerate <500 mg/day (10 g natural protein/day) in order to avoid elevated blood phenylalanine levels. Phenylalanine tolerance does vary between patients depending upon the severity of their disorder and the use of pharmaceutical treatment options such as sapropterin (synthetic tetrahydrobiopterin (BH4), or pegvaliase (phenylalanine ammonium lyase). Sapropterin, an oral drug, is effective in a subset of BH4 responsive patients with PKU and is usually given as an adjunct to dietary treatment [16]. Pegvaliase, delivered by subcutaneous injection, is only licensed for adults with blood phenylalanine levels above the European PKU guidelines target range [17,18]. Neither pharmaceutical treatment option is available via the National Health Service in England.

The additional scrutiny of checking all food ingredient labels for aspartame in food, drinks and drugs intensifies the complexity of management [19]. Aspartame is added to a wide variety of foods: low calorie sweeteners, soft drinks (including fizzy drinks, fruit squashes/cordials), iced tea, flavoured mineral water, energy drinks, dessert mixes, frozen desserts, syrups/dessert sauces, mints, jelly, chewing gum, fruit yogurt, ice lollies, and ice creams. It is also added to around 600 pharmaceutical products (both medically prescribed and over the counter) including chewable multivitamins and cough medications. According to European law, foods containing aspartame must declare it is added either by name or E number (E951) [20]. However, it is not mandatory for manufacturers to state the amount of aspartame added to foods, rendering it impossible for people with PKU to estimate the phenylalanine intake from this source.

A further concern in the UK is the Soft Drinks Industry Levy (SDIL) which was introduced by Her Majesty’s Revenue and Customs (HMRC) in 2018 [21]. It is commonly referred to as the “sugar tax”. This was devised in response to national concerns about rising childhood and teenage obesity and was designed to encourage manufacturers to reduce the added sugar content of their drinks. It is a two-tier levy system: including a standard tax rate applied to drinks with a sugar content between 5 g and <8 g per 100 mL and a higher tax rate applied to drinks with a sugar content ≥8 g per 100 mL. This “sugar tax” has been highly effective with at least 50% of manufacturers reducing the sugar content of their products [22] but it has also led to many manufacturers replacing sugar with artificial sweeteners such as aspartame, potentially marginalising the dietary choices of patients with PKU. A recent equality risk assessment conducted by the HMRC examining the SDIL, stated that they were unaware of any evidence to suggest that the existing warning on food labels about the presence of aspartame in soft drinks was inadequate for people with PKU [23].

It is important to understand the impact of added aspartame to foods, drinks and medications on people with PKU. This paper aims to examine the frequency of accidental aspartame consumption, the reasons for this, and the challenges associated with avoiding aspartame in PKU.

## 2. Materials and Methods

### 2.1. Study Design

We performed a cross sectional online survey. Patients with PKU and/or parents/caregivers of a person with PKU were invited to take part in this study. Respondents were excluded if they did not reside in the UK. The questionnaire was built in the Online Surveys platform (https://www.onlinesurveys.ac.uk (accessed on 1 April 2020)) and placed on the UK National Society for Phenylketonuria (NSPKU) website, with additional promotion on the NSPKU Twitter and Facebook accounts between April and July 2020.

This non-validated questionnaire contained 23 questions; 10 multiple choice (6 of which invited additional comments), 8 multiple response, 3 Likert scale and 2 open-ended questions. A group of experienced research dietitians from Birmingham Women’s and Children’s Hospital (A.P., S.E., A.M.), a colleague at the NSPKU (S.F.) and an expert in survey methodology (M.O.) helped develop the survey with a student dietitian from Birmingham City University (E.N.). The questionnaire was also reviewed by lay people to ensure its readability.

### 2.2. Data Collected

Demographic information was collected about the type of respondent (patient or parent/caregiver of patients aged ≥18 y or <18 y), gender of the person with PKU and confirmation of residency in the UK. Respondents answered questions about any known consumption of foods, drinks and medications containing aspartame, the frequency this had occurred, the reason behind this accidental ingestion and any symptoms this had caused. They were also asked about their knowledge and impact of the sugar tax with respect to the aspartame content of foods and drinks, and the ease of identifying aspartame on food, drinks and medication labels in addition to other challenges of identifying aspartame in products.

Overall themes explored in the survey were: accidental consumption of aspartame in food and drinks, accidental consumption of aspartame in medications, the sugar tax, drinks choice in different venues, label checking, and the effect of aspartame addition on the person with PKU and their family.

### 2.3. Statistics

Quantitative data analysis (inferential and descriptive statistics) was carried out with Statistical Package for the Social Sciences (SPSS) version 25 (SPSS Inc., Chicago, IL, USA). Multiple response questions were analysed with descriptive statistics only. Statistical significance was set at *p* < 0.05.

Qualitative data analyses of open-ended responses were carried out in NVIVO v.12 PRO. The whole survey dataset was imported into NVIVO so that the coding of open-ended responses could be broken down by survey questions including demographic questions. All open-ended responses were analysed thematically.

### 2.4. Ethics

Ethical approval to perform this study (approval number 6085, project title “The accidental consumption of aspartame in PKU: The experiences of patients and their caregivers”) was given by Birmingham City University ethics committee. Adults with PKU and parents/carers of children and adults with PKU gave their consent at the beginning of the online questionnaire. Potential respondents were also advised that data from the survey may be published in an anonymized form. If names or hospitals were mentioned in verbatim abstracts, these were removed from results presented in this manuscript.

## 3. Results

There were 206 wholly or partially completed questionnaires. Fifty-five per cent (*n* = 114) of respondents were adults (18 or over) with PKU or parent/carers of adults with PKU and 45% (*n* = 92) were the parent or carers of children with PKU.

All respondents were normally residents in the UK. The PKU population described by the respondents were: 58% (*n* = 119) female; 41% (*n* = 85) male, 1 respondent was ’non-binary’ and 1 preferred not to say.

### 3.1. Accidental Consumption of Aspartame in Food and Drink

Seventy-four per cent of participants (*n* = 152/206) said that people with PKU had consumed aspartame in a food or drink; 20% (*n* = 42/206) said they had not and 6% (*n* = 12/206) said they did not know.

Of those who had consumed aspartame by accident/error, just under half (47%, *n* = 72/152) said this occurred one to three times; 17% (*n* = 26/152) said 4 to 6 times and 6% (*n* = 9/152) said that it had occurred 7 to 9 times in the last 3 years. One in ten respondents (11%, *n* = 16/152) said that accidental consumption had occurred 10 times or more. Just under one fifth (19%, *n* = 29/152) of respondents could not recount how often accidental consumption had happened. Repeated accidental consumption of aspartame was more frequent in adults with PKU than for children (*p* < 0.0001). In the last 3 years, aspartame had been consumed accidentally 1 to 3 times in 79% (*n* = 42/53) of children and 43% (*n* = 30/70) of adults. In contrast, accidental consumption of 4 to 6 times occurred in 31% (*n* = 22/70) of adults compared to only 8% (*n* = 4/53) in children. Females (79%) with PKU were more likely to report having consumed aspartame than males (67%) (*p* = 0.008, Fisher’s exact test). Eleven per cent (*n* = 8/74) of females had 7 to 9 incidents, compared to 0% (0/48) of males; 18% (*n* = 13/74) of females had 10 or more incidents, 3 times the proportion of males at 6% (*n* = 3/48). Patients that answered “don’t know” were excluded.

The main reasons for accidental consumption of aspartame were manufacturers’ changing product recipes (81%, *n* = 123/152), inability to check the ingredients e.g., drinks purchased in a pub or restaurant or from a vending machine (59%, *n* = 89/152), forgetting to check the label (32%, *n* = 49/152), and picking the wrong product from a shelf when shopping (29%, *n* = 44/152). Other reasons described by the respondents included: served the wrong drink in a bar or restaurant, (*n* = 22), unclear labelling (*n* = 16), not realising a product contained aspartame (*n* = 11), child unsupervised (*n* = 6), or other undefined reason (*n* = 4).

Examples of the verbatim quotes for the 5 most common themes for accidental aspartame consumption.

- “Drinks that were previously free from aspartame and fine to drink had their recipe changed without seemingly advertising the change. This meant that it was only on consumption and tasting the difference from how it used to be that the ingredients were checked, and aspartame was found.”
- “I don’t know how many times I have consumed aspartame, but I know I have. In a crowded bar it is hard to request a specific brand name and it is not possible to read a label on a multi dispensing tap such as that used by bar staff to add coke or tonic to a drink.’’
- “I have never seen a lolly with aspartame in before, so I didn’t check it from the ice-cream man—I checked it only after she had eaten it.’’
- “Both my girls have autism. They do not understand consequences and are unable to challenge/ask people if the drinks contain aspartame, therefore they will just drink what is given to them. They have also picked up the wrong bottles of coke as the packaging is not much different at all’’.
- “Aspartame isn’t required to be listed on alcoholic drinks, therefore it’s hard to know if it’s present or not.’’

### 3.2. Foods/Drinks Involved in Accidental Aspartame Consumption

The food or drinks containing aspartame most reported to be accidentally consumed were fizzy drinks e.g., Coca Cola/lemonade/Irn Bru (68%, *n* = 103/152), fruit squash/cordials e.g., Robinsons Summerfruit squash (40%, *n* = 61/152), chewing gum (30%, *n* = 46/152), flavoured water (25%, *n* = 38/152), ready to drink cartons or bottles of juice/squash e.g., Strawberry Ribena (23%, *n* = 35/152), sports drinks e.g., Lucozade/Powerade (21%, *n* = 32/152), alcoholic drinks (19%, *n* = 29/152), sweets (14%, *n* = 21/152), jelly (9%, *n* = 14/152), tonic water (7%, *n* = 11/152), mints (7%, *n* = 11/152), iced slush drinks (7%, *n* = 10/152), energy drinks e.g., Red Bull (5%, *n* = 7/152), and table top sweetener e.g., Half-Spoon (3%, *n* = 4/152).

### 3.3. Aspartame Consumption of Medically Prescribed and over the Counter Medications

Twenty-three per cent (*n* = 48/206) of responders said that people with PKU had been prescribed medicines by their doctors that contained aspartame. This was more likely to occur in children (30%, *n* = 28/92) than adults (18%, *n* = 20/114).

Seventy-five per cent (*n* = 36/48) said that medicines were not checked by doctors/pharmacists for aspartame, but it was identified by the person with PKU or their carer. Twenty-five per cent (*n* = 12/48) said they had been advised that it was better to take the medicine and not worry about the aspartame content. Four per cent (*n* = 2/48) of respondents said the amount of phenylalanine from aspartame was checked and the number of phenylalanine exchanges adjusted accordingly. Thirteen per cent (*n* = 6/48) gave an “other” response including: ‘was given a replacement medication only after they requested for this to happen’, ‘they accepted the medicine even though they knew it contained aspartame’, were ‘refused an alternative medication’, and ‘health professionals (dispensing the medication) were unaware of aspartame or PKU’. Although most respondents managed to access an alternative suitable medication, it depended on the patient or carer first identifying that aspartame was on the list of ingredients on the original medication.

Most respondents (88%, *n* = 182/206) were aware that some over-the-counter medicines contained aspartame, but 20% (*n* = 37/182) had consumed aspartame from this source.

Some verbatim extracts about the experiences associated with aspartame in medications are given below.

- “I checked the ingredients and found the medicine contained aspartame and had a written warning about phenylalanine. I called the doctor who couldn’t think of a different medicine so was told to go to hospital with my child to receive “better care.”
- “Happens a lot. There have been times when I’ve had to visit several chemists to finally get a variation without aspartame. I’ve also asked the GP to issue a script for an alternative medicine. It’s always down to the patient to check and Drs and pharmacists are unaware.’’
- “Always been told it’s best to take the medication and get better then worry about levels afterwards.’’
- “We checked, and it only had a small amount of aspartame and he was very poorly and he needed to have it.”

### 3.4. “Sugar Tax”

Most respondents (93%, *n* =192/206) were aware of the sugar tax. Many respondents (85%, *n* = 164/192) considered the sugar tax made accidental aspartame consumption more likely (either much more likely, 59% (*n* = 114/192) or slightly more likely, 26% (*n* = 50/192)). Eleven per cent (*n* = 21/192) thought that the sugar tax made no difference to the likelihood of accidental consumption of aspartame and just over 3% (*n* = 6/192) thought that the sugar tax had make it less likely.

Eighty-nine per cent (*n* = 170/192) thought the sugar tax led to fewer choices of drinks and more than two-thirds (68%, *n* = 130/192) considered that drink costs increased. More than four in 10 respondents said that the sugar tax had caused increased stress for the person with PKU and 27%, (*n* = 52/192) reported greater social isolation. Fifteen per cent (*n* = 29/192) of respondents thought that the tax had led to worse blood phenylalanine control for people with PKU. Only 5% (*n* = 10/192) thought the tax had no effect. ‘Other responses’ were commonly expressions of anger, being disheartened or depressed about the situation as the sugar tax increased the burden of dietary treatment even more.

Some examples of verbatim quotes given to the open question responses about the impact of the sugar tax:

- “Drinks are something we can share and enjoy. Drinks that we could enjoy, experiment with, taste and talk about are now becoming less accessible and it has a really big impact on us. Sugar is actually one of the few things that we can ingest without fear of brain damage, and mental and physical damage.”
- “My daughter is aware of the higher cost of the non-aspartame products so will often choose to go without; thinking about the extra expense to us as parents.”
- “It has made an already difficult diet even harder to follow and people just think you are unhealthy choosing sugar versions and a faddy diet.”
- “Soul destroying for a person to check every food label/every morsel they put into their mouths”.

### 3.5. Choice of Drinks in Different Venues

Respondents stated their dissatisfaction with the supply of drinks in different venues (Table 1). This was highest in relation to leisure/sports centres (67%); followed by fast food chains (62%) and restaurants (60%). Forty-nine per cent (*n* = 81/167) were dissatisfied (fairly or extremely) with the choice of drinks in hospitals, when people with PKU attended their clinics. Museums, airports, petrol stations and other people’s homes had some of the lowest dissatisfaction scores but even for these venues, dissatisfaction is high in absolute terms (i.e., there is low satisfaction across all venues and high proportions are neutral on most venues).

### 3.6. Label Checking

Respondents checked labels for food, drinks and medicines most of the time (Table 2). Drink labels (96%, *n* = 196/205) were checked either most of the time or always which is higher when compared with food labels (81%, *n* = 165/203).

Food, drinks and medication labels were always checked more often by parents/caregivers for children. For food, 44% (*n* = 49/111) of adults or carers of adults always checked labels compared with 71% (*n* = 65/92) of parents/carers of children; for drinks, 56% (*n* = 63/113) of adults or carers of adults always checked labels compared with 78% (*n* = 72/92) of parents/carers of children; for medicine, 46% (*n* = 52/112) of adults or carers of adults always checked labels compared with 85% (*n* = 77/91) of parents/carers of children. On average, both adults or carers of adults and carers of children with PKU all checked food, drinks and medicines labels for aspartame ‘most of the time’ but carers of children significantly more so (Table 3).

### 3.7. Ease of Identifying Aspartame on the Ingredient Label

A high proportion of respondents reported it was very easy or fairly easy to identify aspartame on ingredient labels, 63% (*n* = 130/205) for food and 65% (*n* = 133/205) for drinks compared to those who had difficulty, 22% (*n* = 45/205) for food and 23% (*n* = 48/205) for drinks (Table 4). Ease of identification of aspartame on medicines was lower with 46% (*n* = 86/189) reporting it was very easy/fairly easy and 40% (*n* = 76/189) finding it difficult. The number remaining neutral was similar for food, drinks and medication.

### 3.8. Challenges in Identifying Products which Contain Aspartame

The biggest challenges identified by respondents are presented in Table 5 in detail.

Perhaps unsurprisingly, the highest single response category in open-ended responses about the challenges in identifying aspartame is related to product labelling. This was mentioned by nearly half of those who responded to this question.

Verbatim quotes about the challenges relating to identifying aspartame from labels on foods and drinks:

- “Writing is often too small on supermarket products. Ingredients section often very full of text so hard to spot aspartame especially if you are rushing.”
- “Sometimes I find it tricky to identify aspartame in products due to weird E numbers that I have no idea about. Clear labelling of aspartame needs to be on all consumable products.’’
- “If eating out often, the restaurant staff are reluctant to check labels or are unsure about ingredients. Catering size products are not easy for staff to find info. Details of ingredients might only be listed on the outer packaging which may have been discarded.”

Respondents also mentioned that there was no prominent warning about the presence of aspartame or that this information was not consistently in the same place on packaging.

- “You are checking the label for aspartame, but the warning is not always in the same place”.
- “The warning text is very small. It inhibits my son’s independence as it’s unrealistic to expect a child to check for labelling that is so hard to see. After the sugar tax, packaging changed and removed the easy visual clues that you could rely on to indicate that the product had aspartame. As an example, there is now a Coca Cola in a red can which has aspartame in it. There are frequently types with aspartame in and some without with virtually the same packaging, you have to check everything, and this is stressful”.
  Many people commented about the time it takes to check labels.
- “We are really careful when we check labels, but it takes time, and it is difficult sometimes”.
- “I can easily identify aspartame in products with labels, but it is time consuming and annoying. I worry that other caregivers, e.g., grandparents, would not be able to. There is no labelling in restaurants, so we err on the side of caution and only order what we know does not contain aspartame.”

Some respondents suggested that the warning on packaging should be at least as prominent as allergen warnings.

- “Should be written in bold/special box like allergens.”
- “Aspartame should be highlighted in a different colour or bold writing as they do for peanut allergies.”

Overall, 74% (*n* = 152/206) of respondents thought that it would be helpful (fairly or extremely) if manufacturers listed the phenylalanine content of food, drink or medicines on the label. Only 6% (*n* = 13/206) were neutral and 20% (*n* = 41/206) thought it would be fairly or extremely unhelpful.

### 3.9. Effect of Aspartame on People with PKU and Parents/Carers

Table 6 gives the percentage of patients that reported each of the stated effects of aspartame on patients and parents/caregivers managing PKU.

Coding of the open-ended responses about the effect of aspartame showed that the top four themes were: feelings of being different, lack of choice, stress or concern and the additional time required to check all labels. These issues are illustrated in the following verbatim quotes.

- “It’s very isolating for our son. He feels people see him as fussy until we have to explain and even then, they don’t seem to understand.”
- “Feel bad when I can’t find suitable drinks for my children with PKU, whereas my children without PKU can drink whatever they want.”
- “The PKU diet is heavily restricted and time consuming. Aspartame adds another level of restriction and extra time is necessary to check everything before you can buy or eat it.”
- “Checking for aspartame increases stress and anxiety especially when eating out which is supposed to be a nice/happy experience.”

## 4. Discussion

This is the first UK survey to examine the impact of aspartame in food, drinks and medications on people with PKU and their caregivers. We found that repeated accidental aspartame consumption is common, particularly in adults with PKU. Many respondents acknowledged there may be occasions in which aspartame has been inadvertently ingested and there were many concerns about the inability to identify its presence in pre-mixed alcoholic drinks and draft soft drinks in restaurants and bars.

The most unintentionally consumed aspartame containing items included fizzy drinks, fruit squash, cordials, flavoured water, sports drinks and chewing gums. Changes to product recipes, selecting the wrong product when shopping, packaging similarities between aspartame and non-aspartame containing products, unclear labelling, and difficulties identifying aspartame in drinks purchased from restaurants and pubs were commonly identified challenges. This suggests the need for: mandatory ingredient lists for all drinks and foods in restaurants, cafes, bars, and vending machines; distinct front of package labelling when a product recipe has changed; and clear labelling when there are several products within a brand range with some containing aspartame and others not (e.g., Ribena, Fanta, Tango, Robinsons). There should also be mandatory visible “first glance” disclosure of aspartame on packaging. Recently Dutch researchers demonstrated there was wide variability in the aspartame content of soft drinks, particularly the same brand of soft drinks bought in different countries. They have urged European legislators to enforce manufacturers to declare the amount of phenylalanine obtained from aspartame on food and drink labels, so that individuals with PKU are aware of the phenylalanine content of foods and drinks [24]. This ‘call for action’ is supported by NSPKU Medical Advisory Panel of dietitians [25].

Accidental aspartame consumption due to medications occurred in almost a quarter of respondents. Respondents felt there was little awareness or concern about the presence of aspartame in medications amongst medical professionals when they prescribed medication for PKU. Generally, reminders to check prescriptions for aspartame came from patients/parents’ instruction rather than the GP or pharmacist. Aspartame is commonly used as a sugar replacement in antibiotics, chewable tablets and sugar-free liquids. The European PKU guidelines [11,17] recommend that for immediate and short-term treatment of infections, if only aspartame containing medicines are available, it may be better to use these until aspartame-free medication is sourced rather than leave a person with PKU without treatment (for a concurrent illness) as blood phenylalanine levels will rise with infection. However, for chronic long-term use of medications, it is better to find alternative aspartame free medications. Aspartame can be identified from the list of excipients in the medication instruction leaflet or the EMC summary of product characteristics. The amount of estimated phenylalanine in a drug may also be listed and can vary from 1 to 25 mg per dose of medication. There is usually no aspartame warning on the outside packaging of medication and there is no legal obligation to include this [26]. However, it is considered important to have mandatory legislation to identify aspartame on the outer packaging for people with PKU, otherwise it is challenging to recognize its presence at the point of prescription or purchase, and it can be a cause of frustration, inconvenience and distress for carers or people with PKU.

The impact of aspartame in food and drinks on inhibiting socialisation, increasing the incumbrance of dietary management and decreasing autonomy for children and teenagers is evident. Respondents were particularly dissatisfied with the choice of suitable drinks at many venues including fast-food restaurants, leisure centres, tourist venues and even hospital clinics. Respondents were angry that waiters/waitresses or sales vendors convey little understanding or empathy. They were displeased with the lack of aspartame free soft drinks at their hospital, as they considered this to be one location that above all others should demonstrate understanding of their condition. For NHS England hospital trusts, the Commissioning for Quality and Innovation (CQUIN) offer a financial incentive if they provide healthier food and drinks. This includes that 80% of drinks provided/sold must not be sugary. If a hospital trust adheres to the CQUIN for healthy food for NHS staff, visitors, and patients, they receive additional funding worth 0.1% of the trust’s overall budget [27]. Unfortunately, there are no exceptions for vulnerable groups who are unable to tolerate aspartame for medical reasons.

There was much anger and despondency concerning the sugar tax by the respondents to this survey. Although the sugar tax has been implemented to reduce national overweight/obesity, it will not necessarily change unhealthy lifestyle practices. Overall people with PKU and their caregivers felt marginalised by this government policy. The sugar tax has led to diminished choice of favourite branded drinks and increased the cost of sugar containing drinks. For many adults, most available soft drinks in bars now contain aspartame, so the freedom of choice and the ability to enjoy a drink with friends has been withdrawn, which is hard to endure when there are so many other dietary restrictions to contend with. Many people with PKU have a functional approach to food; they eat for necessity rather than pleasure. However, drinking ‘normal’ branded drinks brought normality and choice. Almost 60% of respondents considered that the sugar tax led to more dietary errors and 33% felt fatigued or unwell with aspartame consumption, although no other information was collected about symptoms. Sugar is one of the few foods that is protein free and can be eaten without adversely affecting blood phenylalanine control in PKU. Giving adequate energy intake from very low protein sources is essential to meet energy requirements and to minimise catabolism that can lead to poor blood phenylalanine control [11], so sugar is not an ‘unhealthy’ food for people with PKU when eaten in moderation. Although it is unlikely there will be any reversal of the sugar tax, and it is expected to be extended to other foods, it is disappointing there is little consideration about the impact of the sugar tax on PKU by Public Health England or HMRC. Promoting healthy eating and exercise habits in the general population should be the key to solving obesity rather than focusing on one food component. Taking a balanced approach, offering many healthy choices without compromising the aspartame-free options for people with PKU would be a better policy.

Confusion and regular recipe changes with the addition of aspartame to manufactured foods/drinks affect a child’s ability to self-manage their diet. For foods such as fresh meat, fruit and vegetables there is clear guidance on whether these are either permitted or forbidden in a low phenylalanine diet; but the ingredients, particularly in popular manufactured sweetened products, may change without notice, adding aspartame, with no clear warning to the consumer. It is, therefore, difficult to give pragmatic advice about suitable foods and drinks. Aspartame may be added to many children’s foods such as ice lollies, soft drinks and iced ‘slush’ drinks that may be purchased from an ice cream van or local shop. Consequently, an adult with dietary knowledge should always check the suitability of these foods and the continual checking of food labels is time consuming and endless.

It is incomprehensible that alcoholic beverages with added sweeteners with an alcohol by volume content of 1.2% or more, do not have to declare the type of sweetener on the label. Moreover, legally no nutrition information needs to be supplied on the label of alcohol although appropriate allergen information and relevant quantitative ingredient information should be given [28]. This renders it unmanageable for people with PKU to be confident that any alcoholic drinks with unnamed sweeteners are safe for consumption. Fortunately, it is likely this situation will improve in the next 2 years. A memorandum of understanding (Self-regulatory proposal from the European alcoholic beverages sectors on the provision of nutrition information and ingredients listing) was presented as a joint voluntary commitment to the EU Health Commissioner in June 2019. It committed that by the end of 2022 the list of ingredients on alcohol will be provided according to the EU 1169/2011 law. This law asserts that aspartame should be identified on the list of ingredients and it must state that it contains a source of phenylalanine [29].

There are several limitations to this study. The participants were not randomly selected and individuals without internet access may have been unable to participate. The survey was also promoted on the NSPKU Twitter and Facebook page, meaning participants were more likely to be NSPKU members who may be more proactive and informed about PKU. Therefore, the survey population may not be representative of the entire PKU population for which it is estimated that there are around 2000 UK patients in hospital follow up. Some surveys were completed by caregivers on behalf of patients with PKU and therefore responses to some questions may have been the caregiver’s opinion rather than the actual experiences of those with PKU. It may be that aspartame was consumed more often but respondents did not realise this. Some respondents were unable to remember how many times they had consumed aspartame over the three-year period. The survey was not validated and therefore has not been checked for reliability, however expert opinion was used to develop it. This study should be repeated and expanded in the future using a validated survey that is piloted and carefully applied by health professionals to further improve the accuracy of the data collected.

## 5. Conclusions

It is important that health care professionals and policy makers understand the impact of aspartame and policies affecting the increased use of aspartame such as the sugar tax on the lives of people with PKU. Aspartame addition to food and drinks introduces social constraints, impacts on metabolic control as well as providing a source of frustration, guilt and distress to people with PKU and their carers. It is difficult to adhere to the PKU diet when all ingredients are not readily declared on labels at the point of purchase or issue. This applies to food, drinks, and medicines. It is essential that that industry gives clear and ‘front of package’ labelling about aspartame presence and the amount of phenylalanine that the product contains. Manufacturers should also consider using alternative sweeteners that would be a suitable option for people with PKU.

## Figures and Tables

**Table 1:** Satisfaction with the range of drinks across various venues.

Venue	Extremely Dissatisfied	Fairly Dissatisfied	Neither Dissatisfied nor Satisfied	Fairly Satisfied	Extremely Satisfied
Leisure Centre/Sports Centre (*n* = 165)	*n* = 42 (25%)	*n* = 68 (41%)	*n* = 20 (12%)	*n* = 30 (18%)	*n* = 5 (3%)
Hospital Clinics (*n* = 167)	*n* = 41 (25%)	*n* = 40 (24%)	*n* = 32 (19%)	*n* = 47 (28%)	*n* = 7 (4%)
Fast Food Chains (*n* = 193)	*n* = 47 (24%)	*n* = 73 (38%)	*n* = 23 (12%)	*n* = 41 (21%)	*n* = 9 (5%)
Pubs/Bars (*n* = 188)	*n* = 42 (22%)	*n* = 69 (37%)	*n* = 13 (7%)	*n* = 59 (31%)	*n* = 5 (3%)
Restaurants (*n* = 195)	*n* = 41 (21%)	*n* = 76 (39%)	*n* = 26 (13%)	*n* = 46 (24%)	*n* = 6 (3%)
Schools (*n* = 123)	*n* = 24 (20%)	*n* = 42 (34%)	*n* = 24 (20%)	*n* = 26 (21%)	*n* = 7 (6%)
Tourist Attraction e.g., Alton Towers (*n* = 170)	*n* = 32 (19%)	*n* = 65 (38%)	*n* = 26 (15%)	*n* = 40 (24%)	*n* = 7 (4%)
Motorway Cafes (*n* = 170)	*n* = 31 (18%)	*n* = 58 (34%)	*n* = 28 (16%)	*n* = 42 (25%)	*n* = 11 (6%)
Cafes (*n* = 197)	*n* = 34 (17%)	*n* = 63 (32%)	*n* = 32 (16%)	*n* = 63 (32%)	*n* = 5 (3%)
Hotels (*n* = 170)	*n* = 29 (17%)	*n* = 55 (32%)	*n* = 36 (21%)	*n* = 43 (25%)	*n* = 7 (4%)
Workplace (*n* = 113)	*n* = 19 (17%)	*n* = 30 (27%)	*n* = 25 (22%)	*n* = 29 (26%)	*n* = 10 (9%)
College (*n* = 64)	*n* = 10 (16%)	*n* = 23 (36%)	*n* = 17 (27%)	*n* = 13 (20%)	*n* = 1 (2%)
Airports (*n* = 165)	*n* = 24 (15%)	*n* = 45 (27%)	*n* = 38 (23%)	*n* = 43 (26%)	*n* = 15 (9%)
Nurseries (*n* = 73)	*n* = 10 (14%)	*n* = 21 (29%)	*n* = 22 (30%)	*n* = 17 (23%)	*n* = 3 (4%)
Petrol Stations (*n* = 180)	*n* = 23 (13%)	*n* = 49 (27%)	*n* = 25 (14%)	*n* = 64 (36%)	*n* = 19 (11%)
University (*n* = 65)	*n* = 7 (11%)	*n* = 24 (37%)	*n* = 18 (28%)	*n* = 13 (20%)	*n* = 3 (5%)
Other People’s Homes e.g., Friends/Family (*n* = 198)	*n* = 12 (6%)	*n* = 61 (31%)	*n* = 42 (21%)	*n* = 65 (33%)	*n* = 18 (9%)

Abbreviations: n: number of respondents. This varies considerably and is low for some venues such as ‘university’ and ‘nurseries’ because these are used predominantly by particular demographic groups and those that did not use them chose ‘not applicable’ and did not rate.

**Table 2:** Proportion of respondents who check food, drinks and medicine labels.

	Not at All	Rarely	Sometimes	Most of the Time	Always
Food (*n* = 203)	*n* = 1 (<1%)	*n* = 13 (6%)	*n* = 24 (12%)	*n* = 51 (25%)	*n* = 114 (56%)
Drinks (*n* = 205)	*n* = 0 (0%)	*n* = 0 (0%)	*n* = 9 (4%)	*n* = 61 (30%)	*n* = 135 (66%)
Medicines (*n* = 203)	*n* = 8 (4%)	*n* = 18 (9%)	*n* = 18 (9%)	*n* = 30 (15%)	*n* = 129 (64%)

**Table 3:** Mean levels of checking food, drink and medicine labels by age group.

	Adult (18 or Over) with PKU or Parent/Carer of Adult with PKU (Mean), *n* = 114	Parent or Carer of Child with PKU (Mean), *n* = 92	Total (Mean), *n* = 206	Mann Whitney Test *p* Value
Food	4.07	4.58	4.30	*p* < 0.001
Drinks	4.50	4.75	4.61	*p* < 0.001
Medicines	3.86	4.74	4.25	*p* < 0.001

Abbreviations: PKU, Phenylketonuria; n: number of respondents. The mean values relate to a scale of 1 to 5 (1 = Not at all; 2 = Rarely; 3 = Sometimes; 4 = Most of the time; 5 = Always).

**Table 4:** Perceived ease of label checking by product type.

	Very Difficult	Fairly Difficult	Neither Difficult nor Easy	Fairly Easy	Very Easy
Food (*n* = 205)	*n* = 11 (5%)	*n*= 34 (17%)	*n* = 30 (15%)	*n* = 89 (43%)	*n* = 41 (20%)
Drinks (*n* = 205)	*n* = 8 (4%)	*n* = 40 (20%)	*n* = 24 (12%)	*n* = 80 (39%)	*n* = 53 (26%)
Medicines (*n* = 189)	*n* = 19 (10%)	*n* = 57 (30%)	*n* = 27 (14%)	*n* = 57 (30%)	*n* = 29 (15%)

**Table 5:** Challenges in identifying products which contain aspartame.

Challenges Faced in Identifying If a Food, Drink or Medicine Contains Aspartame	Percentage Responses (%)	Number of Respondents Per Total Sample (*n* = 206)
Difficulties in Identifying Aspartame in Food or Drinks Consumed in Restaurants, Pubs, Cafes, Vending Machines	77	158
Similarities in Appearance of Non-Aspartame and Aspartame Containing Products	62	127
Time Taken to Identify if a Product Contains Aspartame	56	115
Unclear Labelling	55	114
Easy to Make Mistakes	44	91
Unable to Read the Writing on Food Labels (Writing too Small, too Shiny)	42	87
Lack of Knowledge about which Products Contain Aspartame	20	42
Have no Challenges	4	8
Other	3	7
Don’t Know	<1	1

**Table 6:** Reported effects of aspartame on the person with PKU and parent/carer.

Effects of Aspartame	Percentage Responses (%)	Number of Respondents Per Total Sample (*n* = 206)
Limits Suitable Drinks in Restaurants/Pubs/Cafes	86	178
Increases Time taken to do Food Shopping	80	164
Causes Anxiety for Person with PKU	52	106
Causes Anxiety for Parent/Carer	46	95
Causes Guilt for Parent/Carer	42	87
Causes Social Isolation	42	87
Person with PKU unable to buy Food or Drinks from Shops, Causing Loss of Independence	40	83
Have to Keep Food Products Separate in the House between PKU and Non-PKU Products	36	75
Causes Person with PKU to Feel Unwell	33	68
Causes Guilt for Person with PKU	33	67
Has no Effect	5	11
Other	4	8

Abbreviations: PKU: Phenylketonuria.

## Data Availability

The data will be made available from the authors upon reasonable request.
